# Argyrophilic grain disease: an update about a frequent cause of dementia

**DOI:** 10.1590/S1980-57642009DN30100002

**Published:** 2009

**Authors:** Lea T. Grinberg, Helmut Heinsen

**Affiliations:** 1 MD, PhD, Department of Pathology, University of São Paulo Medical School, São Paulo, SP, Brazil.; 2 MD Labor fuer Morphologische Hirnforschung der Klinik und Poliklinik fuer Psychiatrie und Psychotherapie, University Of Wuerzburg, Wuerzburg, Germany.

**Keywords:** pathology, brain, neurology, argyrophilic grain disease, tau

## Abstract

Argyrophilic grain disease (AGD) is a sporadic, very late-onset tauopathy, accounting for approximately 4–13% of neurodegenerative dementias. AGD may manifest with a range of symptoms such as cognitive decline and behavioral abnormalities. To date, no study has been able to demonstrate a distinct clinical syndrome associated with AGD. The diagnosis is exclusively based on postmortem findings, the significance of which remains controversial because up to 30% of AGD cases are diagnosed in subjects without any cognitive impairment, while AGD findings often overlap with those of other neurodegenerative processes. Nevertheless, the presence of AGD is likely to have a significant effect on cognitive decline. The neuropathological hallmarks of AGD are argyrophilic grains, pre-neurofibrillary tangles in neurons and coiled bodies in oligodendrocytes found mainly in the entorhinal cortex and hippocampus. This review aims to provide an up-to-date overview of AGD, emphasizing pathological aspects. Additionally, the findings of a Brazilian case series are described.

## Abstract (pt)

Doença com grãos argirofílicos (DGA) é uma taupatia, não-familial, de início tardio que corresponde a cerca de 4 a 13% das demências neurodegenerativas. DGA se manifesta com uma gama de sintomas como déficit cognitivo e distúrbios comportamentais. Até o momento, não se conhece nenhuma característica clínica que distinga DGA de outras demências. O diagnóstico é baseado nos achados anatomopatológicos, cujo significado é controverso, quer porque cerca de 30% dos casos de DGA não apresentam sintomas, quer porque DGA é freqüentemente encontrada junto a outras alterações neurodegenerativas. Entretanto, acredita-se que a presença de DGA impacte a cognição. Os marcadores neuropatológicos de DGA são: grãos argirofílicos, pré-emaranhados neurofibrilares e corpúsculos em forma de vibrião em oligodendrócitos. As áreas mais afetadas são o córtex entorrinal, hipocampo e estruturas límbicas. Essa revisão almeja sumarizar o conhecimento mais atual sobre DGA e descrever brevemente achados de uma casuística brasileira.

## Introduction and historical background

Argyrophilic grain disease (AGD) is a very late-onset tauopathy, accounting for approximately 4–13% of neurodegenerative dementias.^1-5^ The name AGD stems from the argyrophilic structures characteristic of this entity.

AGD was first described in 1987 by Braak and colleagues as a distinctive degenerative disease characterized by argyrophilic grains confined to limbic structures affecting a subset of patients with adult onset dementia.^6^

Although highly prevalent, to date no study has been able to demonstrate a distinct clinical syndrome associated with AGD and only a few series have described clinical features that may correlate with the presence of this entity.^7-12^

The diagnosis is based solely on postmortem findings. The impact of the grains is controversial for two main reasons. Firstly, up to 30% of the AGD cases are diagnosed in subjects without any cognitive impairment.^8,12^ Secondly, AGD findings typically overlap with other neurodegenerative findings in cognitively impaired subjects, especially neurofibrillary tangles (NFT), one of the hallmark lesions of Alzheimer’s disease.^8,9,13-16^ The objective of this review was to provide an up-to-date overview of AGD and to describe the findings of a Brazilian case series drawn from the Brain Bank of the Brazilian Aging Brain Study Group (BBBABSG).

## Clinical symptoms

AGD may manifest with a range of symptoms including cognitive decline, dementia^4,7,15,17^ and behavioral abnormalities.^7,11,18,19^

Amnestic cognitive impairment tends to be mild and non-progressive.^9,20^ A recent study verified that AGD patients retain abilities in verbalizing and articulating as well as problem-solving skills, on average, for approximately 2 years longer than Alzheimer’s disease (AD) patients. However, there is no distinctive clinical profile for evaluating single cases.^21^

AGD may occasionally present as frontotemporal dementia, and is considered one of the possible neuropathological entities underlying frontotemporal dementia.^22,23^

Although the commonly associated AD pathology makes it difficult to assign specific clinical symptoms to AGD, the presence of AGD has a significant effect on cognitive decline; e.g. demented with AGD display considerably less AD-associated pathology than pure AD would show at the same clinical stage.^24,25^

In summary, a precise test for clinical diagnosis of AGD has yet to be developed.

## Neuropathological aspects

Gross examination of the brain shows moderate to severe cerebral atrophy with average brain weight of 1084±109g up to 1120g.^16^

The neuropathological hallmarks of AGD are argyrophilic grains, pre-neurofibrillary tangles in neurons (pre-tangle neurons) and coiled bodies in oligodendrocytes. Given that all of these hallmarks are phospho-tau positive, AGD is classified as a tauopathy.

## Argyrophilic grains (AGs)

**Figure 1:**
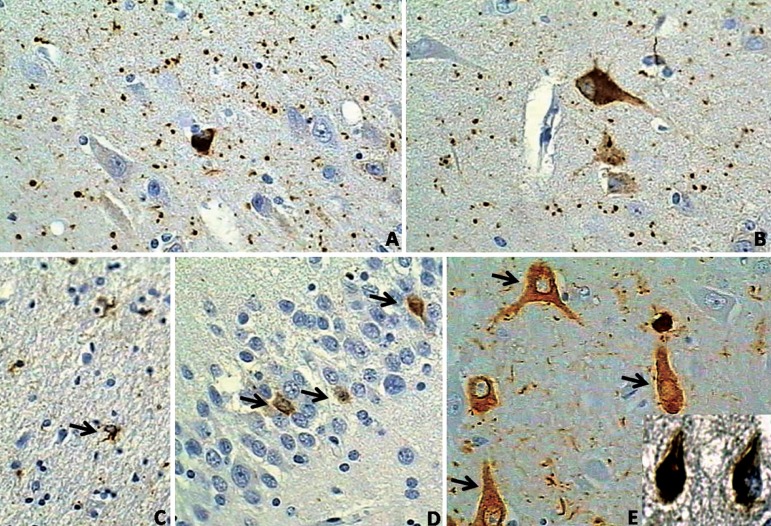
Neuropathological features of argyrophilic grain disease. All the histological slides are immunostained with PHF-1 antibody against phospho-tau. (A) Argyrophilic grains spread in the neuropil of region CA1 of the hippocampus. 400×. (B) The same as in A. Note a pre-tangle in the picture. 400×. (C) Coiled body (arrow) in an oligodendrocyte in the white matter adjoining the entorhinal cortex. 400×. (D) Pre-tangles in the dentate gyrus (arrows). 400×. (E) Pre-tangles in the CA1 region of the hippocampus. Note the difference of the phospho-tau aspect between pre-tangles (diffuse) and the tangles (neurofibrillary) to the bottom right.

The term is derived from their strong staining using the Gallyas silver iodide method. However, it is noteworthy that AGs are not stained by all silver methods,^26^ indicating that AGs have specific features. AGs are also labeled using immunohistochemistry against phospho-tau protein, such as PHF-1 and AT^8^ antibodies (Figure 1A,B).

AGs occur mainly in transentorhinal, and entorhinal cortex, the CA1 area of the hippocampus and presubiculum. It is important to notice that these areas are also affected early by phospho-tau changes in AD. The adjoining temporal cortex, orbitofrontal cortex, insular cortex, basolateral nuclei of the amygdala and hypothalamic lateral tuberal nucleus can also be involved.^13,27^ The source of AGs probably lies in pre-tangle projection neurons found in the same location as the AGs^1,4^. AGs are predominantly localized in dendrites and dendritic branches^1,27,28^, although association of AGs with axons has also been reported.^4^

AGs are small, about 4–8 micrometer, spindle shaped, rod-like, button-like or round bodies in the neuropil (Figure 1). Ultrastructurally, AGs contain straight filaments or tubules measuring 9–25 nm.^13^

## Pre-tangle neurons (Figure 1B,D,E)

Pre-tangle neurons are a constant finding in AGD, and their regional distribution is the same as that for AD. They are also found in the dentate gyrus (Figure 1D).^4-6^ Pre-tangle neurons in AGD do not apparently differ from pre-tangle neurons in AD^29,30^ (Figure 1B,D).

## Coiled bodies in oligodendrocytes

Although being invariably found in AGD, coiled bodies are similar to those observed in many other tauopathies and therefore lack specificity.^31,32^ (Figure 1C).

## Other findings

***Tau-containing astrocytes*** – Astrocytes containing phospho-tau show granular immunoreactive cytoplasm rather than dense inclusions akin to those seen in tufted astrocytes in progressive supranuclear palsy. Generally, they appear in clusters, thus being suggestive of plaques seen in corticobasal degeneration. The presence of tau-containing astrocytes is variable from one case to another, and when found are usually confined to the limbic system.

***Ballooned neurons*** – A-β-crystallin-positive ballooned neurons are commonly observed in the amygdala, in the presubiculum and middle layers of the basal temporal cortex in AGD.^33^ Yet ballooned neurons are usually interpreted as non-specific lesions, given these are a common finding in many familial and sporadic tauopathies and AD.^34,35^

Tangles and neuropil threads – Variable numbers of tangles and neuropil threads may be present in the same regions as in AD. This has caused some confusion about the borderline between AGD with a few tangles and AGD with associated AD.^36^ Most pathologists categorize AD changes (neurofibrillary tangles and neuropil threads) in AGD according to the guidelines of Braak and Braak.^37^

In their own case series Braak and colleagues classified most of the AGD cases as having AD ranging from stage from I to IV.^18^ However, the apparently small percentage of AGs in advanced stages of AD must be interpreted with care, as the substantial phospho-tau-immunoreactive pathology in such cases may incrementally hamper the visualization of AGs. Recent studies using 4R tau-specific antibodies which highlight AGs, have shown a higher prevalence of AGs in advanced stages of AD.^38^ Nevertheless, AGD is usually not accompanied by substantial β-amyloid deposits.^39^

***Staging of AGs*** – In 2004, Saito and colleagues proposed a staging system for AGD based on a refined analysis of a large series.^2^ This system presumes an antero-posterior progression of the disease. Rare cases have shown widespread AGs throughout the temporal lobe, limbic system, frontal cortex and brain stem.^40-42^ An up-dated staging system was proposed by Ferrer and colleagues in 2006.5 This recent systematic staging of AGs does not include accompanying changes. Table 1 compares the two staging systems.

**Table 1:** Comparison of the two neuropathological staging systems for argyrophilic grain disease, as proposed by Saito et al. in 2004 and Ferrer et al. in 2008.

Staging system	Stage			
	I	II	III	IV
Saito et al., 2004^2^	Ambient gyrus and its vicinity	I + anterior and posterior medial temporal lobe, including the temporal pole, as well as the subiculum and entorhinal cortex	II + septum, insular cortex and anterior cingulate gyrus, and spongy degeneration of the ambient gyrus	Moderate to severe additional involvement of the neocortex and brainstem
Ferrer et al., 2008^5^	Anterior entorhinal cortex; mild involvement of the cortical and basolateral nuclei of the amygdale and of the hypothalamic lateral tuberal nucleus	more severe involvement of the nuclei involved in stage I + Entorhinal and transentorhinal cortices; anterior CA1	II + mild involvement of CA2, CA3, presubiculum; other nuclei of the amygdala; dentate gyrus, other nuclei of the hypothalamus, temporal, orbitofrontal and insular cortices, cingulated gyrus, ncl. accumbens, septal nuclei; midbrain	

## Biochemistry of tau in AGD

Tau proteins are encoded by the tau gene on chromosome 17. Alternative splicing of exons 2, 3 and 10 results in six isoforms, which in turn give rise to six different mRNAs.

Tau proteins resulting from encoding exon 10 have four repeat regions (4R tau), whereas those lacking encoding exon 10 have three repeat regions (3R tau).^43,44^

The function of tau largely depends on post-translational modifications including phosphorylation and dephosphorylation, a balanced action between protein kinases and protein phosphatases. Several kinases have been implicated in tau phosphorylation.^45-50^

In contrast to AD, in which 3R tau and 4R tau forms are found, AGD is characterized by a double band of 68 and 64 kDa similar to that found in progressive supranuclear palsy and corticobasal degeneration. Therefore, AGD is considered a 4R tauopathy. The use of specific anti-4R antibodies has corroborated this biochemical observation.^51^

Interestingly, the occurrence of tangles and pre-tangles in the hippocampal CA2 area, a very common finding in AGD, is associated with 4R tauopathy.^52^

## Genetics

AGD appears to be sporadic given that a familial form has yet to be reported. The tau gene or microtubule-associated protein tau (MAPT) locus is located on chromosome 17q21.^53^ The region is divided into two predominant haplotypes, H1 and H2. In 2008, a single case with AGD phenotype was linked to a novel S305I MAPT mutation and^54^ there is evidence from one series that the incidence of MAPT H1 is slightly higher in AD cases with AGD than in those without AGD.^38^ However, other genetic studies have failed to discover a sustained link between AGD and a particular gene locus. The frequency of apolipoprotein E e4 (ApoE e4) allele, the most important genetic risk for AD, proves similar to that of the general population in cases of AGD.^55^ Nevertheless, the frequency of ApoE e2 is higher in AGD than that observed in both AD or controls.^51,56^

## Differential diagnosis

Neuropathological studies have shown frequent association of AGD with other neurodegenerative diseases, the most common being AD. AGD has also been reported together with other tauopathies, Creutzfeldt-Jakob disease, a-synucleinopathies and hippocampal sclerosis.^8,16,18,57-60^

## AGD in the case series from the Brain Bank of the Brazilian Aging Brain Study Group

In the BBBABSG series, AGD was diagnosed in 36 (11.5%) out of the first 307 fully analyzed cases. In accordance with other series, AGD was more frequently found in older subjects (*p*<0.05). No statistically significant difference was found concerning gender, years of schooling, cognitive status, Braak and Braak neurofibrillary stage, presence of β-amyloid plaques or Lewy bodies among the cases with and without AGD. Most interestingly, AGD was the only finding in 14.3% of the subjects manifesting moderate or severe parkinsonism signs. Although AGD is not classically associated with parkinsonism, we are not the first to report this association.^61^ AGD is usually associated with finding of allocortical neurofibrillary tangles. Accordingly, in our series only two AGD cases (6.9%) were devoid of tangles. One of these subjects, a 79-year-old male had no cognitive decline, whereas the other subject, a 82-year-old female showed severe dementia, interpreted as being attributed to the severe burden of microvascular changes and lacunes rather than the presence of AGs.

## Conclusions

AGD is a sporadic and distinct tauopathy often found in the brain of older subjects. Although linked to cognitive decline, behavioral problems and even parkinsonism, no study to date has demonstrated any clinical or laboratory particularity able to distinguish AGD from other neurodegenerative diseases, while several subjects harboring AGD appear not to be demented. In recent years, studies based on well-conducted clinicopathological correlation series have pointed to older age as the only risk factor for AGD, and revealed that AGD may lower the threshold for dementia. Neither of these findings was observed in our series.

Several points still remain obscure. What is the origin of the grains? Is AGD a distinct clinical syndrome? How can neurofibrillary tangles of Alzheimer disease be differentiated from those found in AGD? Is there any hallmark clinical symptom suggestive of the presence of AGs in the brain? Additional comprehensive, prospective clinicopathological correlation studies are required to answer many of these questions.
